# Lecture, model, or both? Optimizing fracture education with 3D printing: a longitudinal interventional study

**DOI:** 10.1186/s41205-026-00342-w

**Published:** 2026-08-25

**Authors:** Christopher Cramer, André Strahl, Karl-Heinz Frosch, Miriam Rüsseler, Tobias Dust

**Affiliations:** 1 Department of Trauma Surgery and Orthopedics, University Medical Center Hamburg-Eppendorf (UKE), Martinistraße 52, 20246 Hamburg, Germany; 2 Department of Psychosomatic Medicine and Psychotherapy, University Medical Center Hamburg-Eppendorf (UKE), Martinistraße 52, 20246 Hamburg, Germany; 3 Institute for Medical Education and Clinical Simulation, Faculty of Medicine, Goethe University, Theodor Stern Kai 7, 60590 Frankfurt/Main, Germany

**Keywords:** 3D printing, Medical education, Teaching methods, Fracture education, Trauma surgery

## Abstract

**Background:**

Traditional anatomical training methods in surgery, like cadaver dissection and didactic lectures, face limitations in availability, cost, and translating 2D to 3D understanding. Three-dimensional (3D) printing offers an innovative solution for medical education, particularly in orthopedics and trauma surgery, where complex bone structures are crucial. 3D-printed models show promise for improving anatomical learning, spatial understanding, and student satisfaction.

**Methods:**

This longitudinal interventional study investigated the impact of different teaching methods on medical students’ knowledge acquisition and perceptions regarding fibula fracture management. Sixty-seven 3rd or 4th-year students, randomized into three groups, participated. Group 1 received a 20-minute lecture, Group 2 engaged in model-based learning using a 3D-printed fibula model, and Group 3 received a combination of lecture and model-based learning. Knowledge was assessed via a written quiz, and subjective feedback and personal motivation were measured. Assessments occurred immediately before (t0), immediately after (t1), and four weeks after (t2) the teaching unit.

**Results:**

At baseline (t0), there were no significant group differences in processing time. Immediately post-training (t1), the lecture group showed the shortest processing time. The lecture + practice group achieved the highest mean knowledge score at t1, significantly outperforming both practice and lecture groups (8.25 vs. 5.09 vs. 6.82, *p* < 0.01). At the four-week follow-up (t2), significant group differences persisted, with lecture + practice maintaining a significantly higher mean score than practice (5.75 vs. 3.46, *p* < 0.05). Overall, knowledge significantly improved from t0 to t1 and t0 to t2, though a decrease was noted between t1 and t2. Subjective feedback consistently indicated that practical and combined methods were more suitable for understanding, enhanced security, and promoted interest.

**Conclusions:**

3D models improve anatomical learning and student satisfaction. The combined lecture + model approach demonstrated superior immediate and sustained knowledge acquisition for complex topics like fracture management. This aligns with pedagogical theories such as constructivism and cognitive load theory, suggesting that integrating theoretical context with physical models optimizes learning outcomes. This study underscores the importance of rigorous comparative designs for effectively integrating innovative tools like 3D printing into medical education.

**Clinical trial number:**

Not applicable.

## Background

With the rapid emergence of 3D printing technology (3DP), surgeons have recently begun to utilize and implement it in nearly all areas of surgery [1, 2]. Applications range from anatomical models intended primarily for surgical planning to surgical guides and implants that have potential value in clinical practice [1–3].

3D-printed bone models are highly accurate in terms of morphology, including the texture of bone fractures [4]. With continuous development, 3DP technology has now reached a reasonable price and production time [2] and is finding its way into medical education [1, 3].

The further development of various printable materials (skin, connective tissue, muscles, vascular systems, and bones) in combination enables realistic simulation of entire body regions in their properties, haptics, and surgical access possibilities [5, 6].

Current training concepts for young surgical residents and medical students offer only limited access to cadaver models as part of clinical training due to the elaborate preparation required. In addition, a wide range of pathologies is required to ensure a learning effect – conditions that are severely limited in times of financial and time constraints [7].

The use of simulations in surgical training and medical education has been thoroughly researched, and the benefits of simulations are well known [6]. 3D-printed models have been shown to be clearly superior to virtual 3D models, as students and residents report greater enjoyment, a higher personal learning effect, and more confidence in explanations to colleagues [1]. Although the introduction of 3D-printed models in surgery is still in its infancy, several studies on 3D-printed simulation models in the field of neurosurgery and cardiothoracic surgery have shown remarkable results [5, 6]. In contrast, there is still little literature in the field of orthopedics and trauma surgery on the benefits of 3D printing technology in anatomical training, surgical training, and preoperatively assisted surgical training.

The study of human medicine includes numerous lectures dealing with a wide range of topics – from theoretical foundations to practical issues relevant in surgery. In the field of orthopedics and trauma surgery in particular, manual activity is indispensable. These skills and abilities are learned and consolidated through repetitive measures (especially in the operating room) and are stored in long-term memory so that they can be retrieved and applied at any time. However, this practical training is neglected during studies, so that this field cannot be adequately represented with the traditionally used learning methods. Furthermore, the theoretical knowledge transfer is not sufficiently linked to long-term knowledge retention and must be adapted to the actual requirements. A third point, which is crucial, is that motivation has a considerable share in medium- to long-term knowledge retention. By using practical fracture models, this can be awakened in students and the interest in orthopedics and trauma surgery can be consolidated in the long term.

Our working group, an interdisciplinary team of surgeons and engineers, has been intensively involved with the possibilities of 3D printing in medical practice for years. In several studies [8–10], we have already demonstrated how three-dimensional models, based on patient CT data, improve preoperative planning and intraoperative procedures. For example, we were able to show that complex fractures can be better understood through the haptic experience on the model and the necessary surgical steps can be planned more precisely. The three-dimensional models allow surgeons to literally “grasp” the patient’s anatomy, allowing them to mentally rehearse the operation in advance. This not only leads to an improved surgical strategy, but also to a reduction in operating time and the risk of complications. Our previous studies have focused primarily on the further training of surgical residents.

In parallel, we came across two interesting studies [11, 12] that highlighted the deficits in the practical training of medical students, particularly in the field of orthopedics and trauma surgery. The authors of this study argued for a stronger integration of practical learning methods to promote anatomical understanding and manual skills of students. In this regard, it could be shown that practical content and knowledge transfer alone do not achieve long-term knowledge gain, but the motivation of students can be positively influenced to a considerable extent [13]. Surgical learning, however, requires a complex interplay of cognitive and psychomotor skills. It has been shown that there is a need to align training with the individual needs of learners, taking into account their level of experience. Technology can support surgical learning by enabling different learning modalities such as videos, simulations, 3D printing, and virtual reality. However, the authors emphasize that technology must be used sensibly and purposefully so as not to increase the cognitive load of learners [12]. 

Inspired by these findings and driven by the desire to improve medical education, the idea for our project was born. We wanted to use the advantages of 3D printing to give students early access to orthopedics and trauma surgery in a practical way.

## Materials and methods

**Fig. 1:**
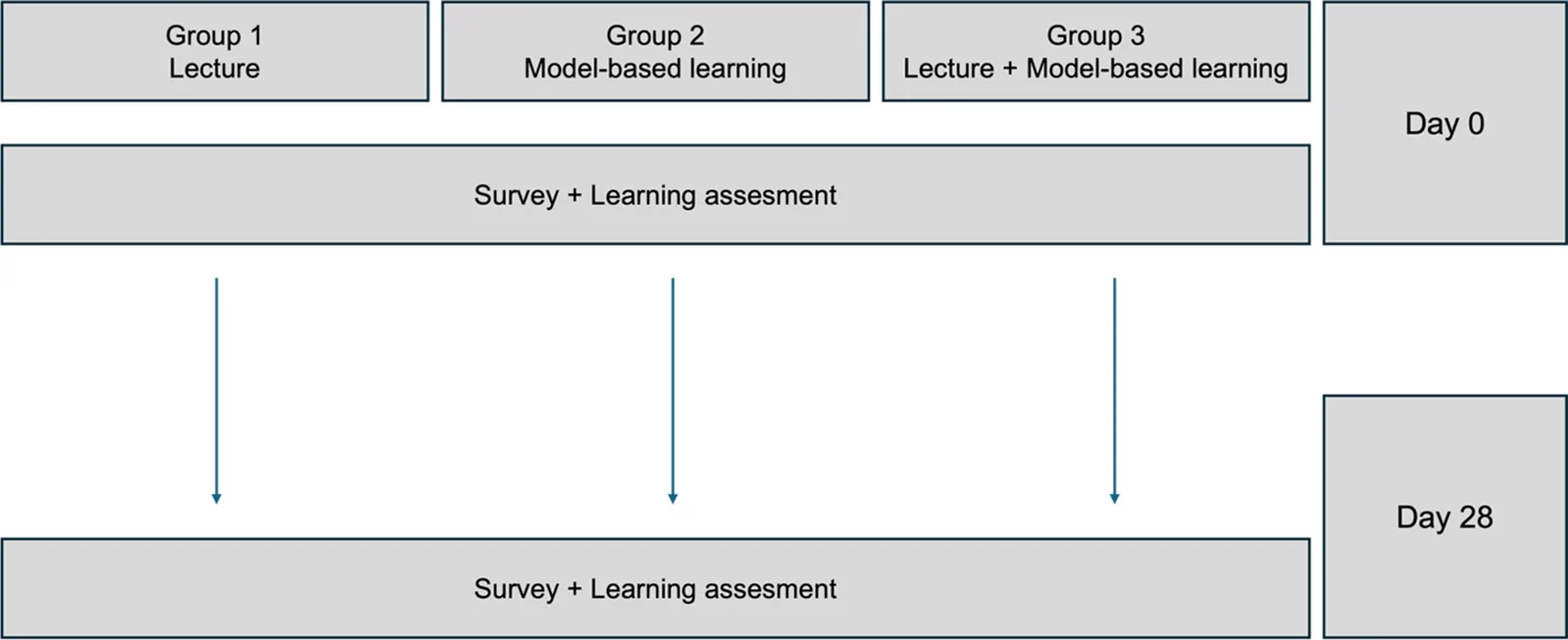
Schematic study procedure: Three groups were randomized into different learning units: lecture, model-based, or lecture + model-based. Tests were performed immediately before and after the learning unit, with a third follow-up test four weeks later

Three groups of students were formed. The allocation to these groups was randomized. All participating students were in their 3rd or 4th year of study, and all had already passed the exam in trauma surgery. A total of 67 students were included for this purpose (Group 1: 24, Group 2: 16, Group 3: 27) (Fig. 1).

**Fig. 2:**
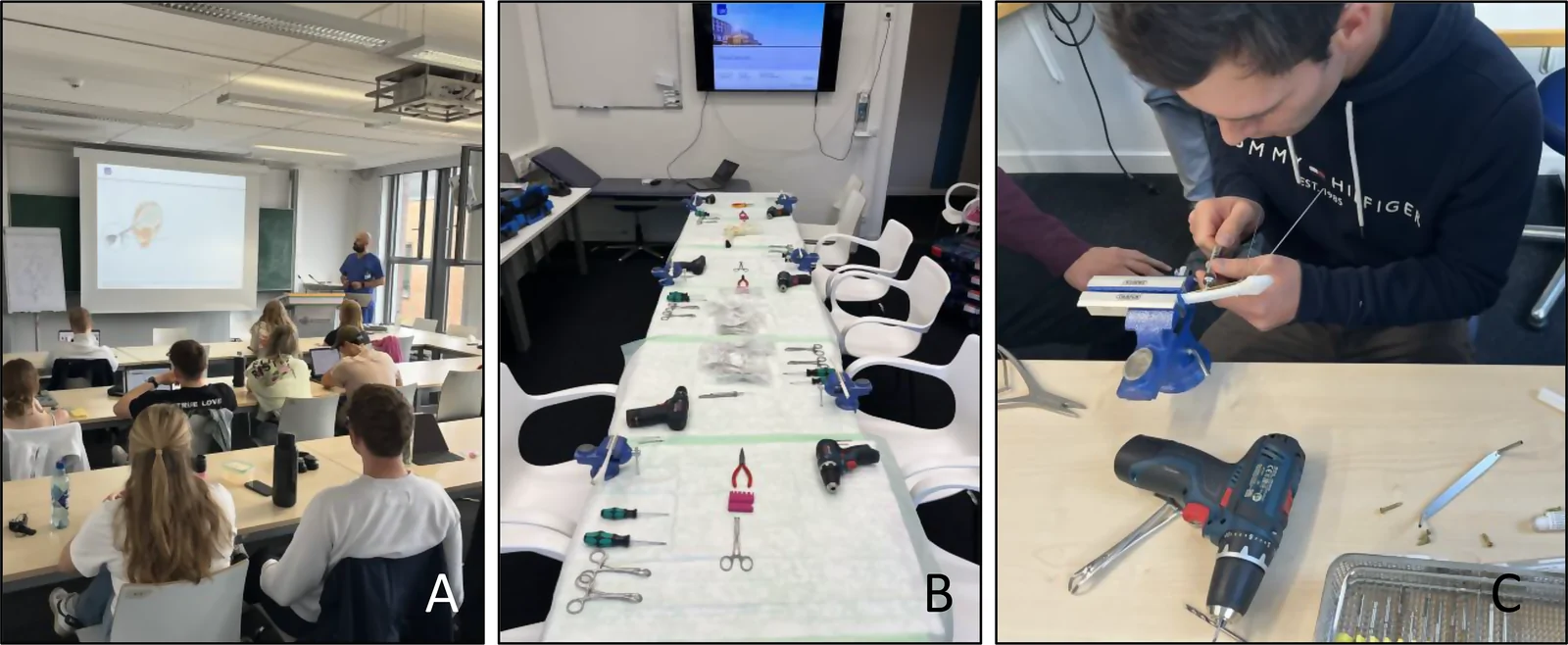
Teaching group during a theoretical lecture on the surgical management of a distal fibula fracture (**A**); prepared workstations for the practical training session (**B**); medical student performing osteosynthesis of a distal fibula fracture using a one-third tubular plate on a 3D-printed fracture model (**C**)

**Fig. 3:**
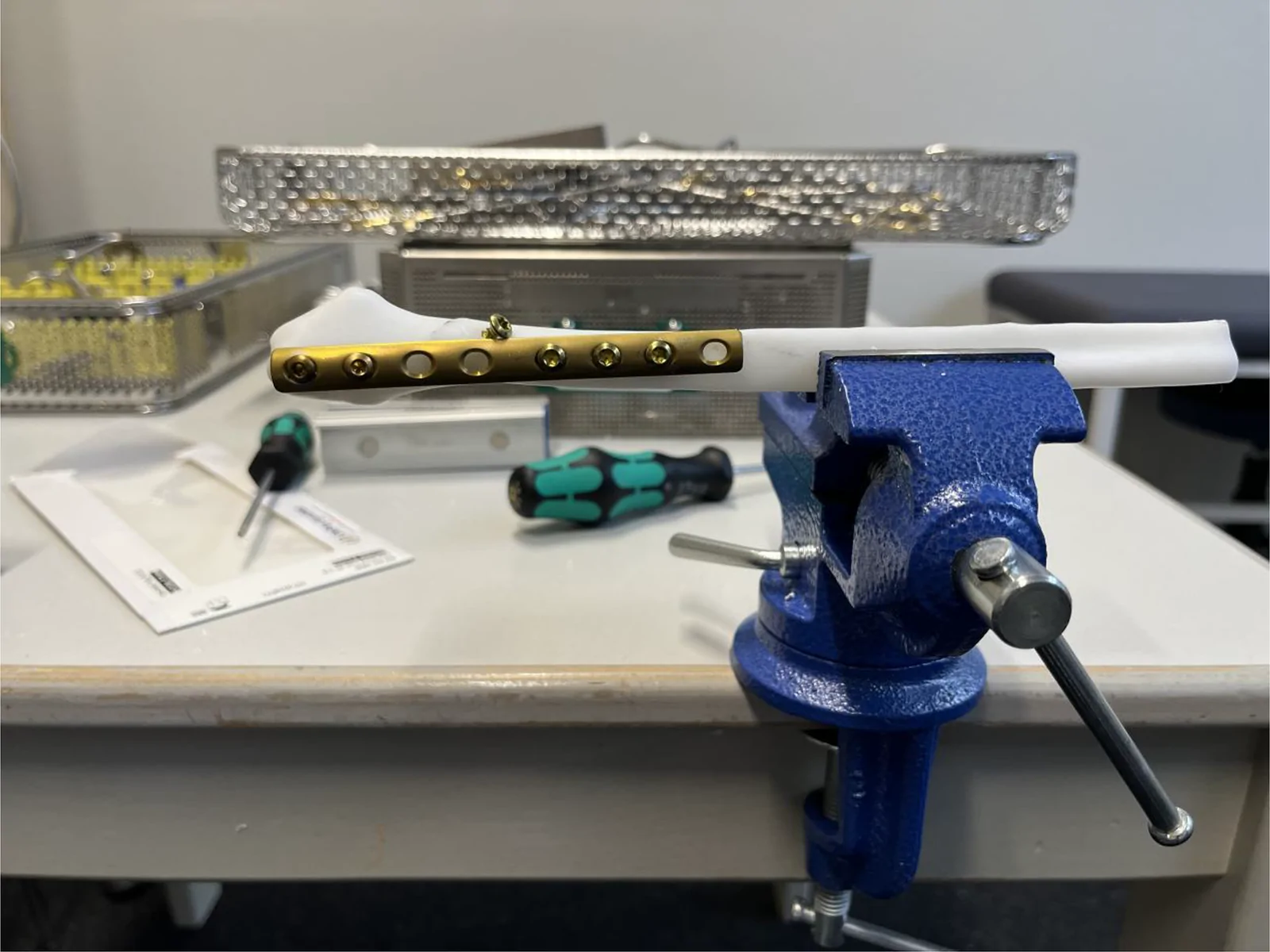
Result of a Weber B fracture osteosynthesis using a one-third tubular plate and lag screw fixation

Group one and group three received a 20-minute lecture on the management of fibula fractures (Fig. 2A). This covered both general fracture theory and specific techniques of surgical management (reduction tools, principle of lag screws, etc.). This lecture was standardized and was always held by the same attending surgeon, ensuring consistency across groups. Group two and group three (+ lecture) learned these contents in a practical manner using a model (Peyton’s Presentation) (Fig. 2B, C) [14]. Here too, the procedure was standardized with clear explanations and the use of identical materials. In both groups, participants were tasked with performing a surgical fixation of a pre-fractured distal fibula using a lag screw and one-third tubular plate on a 3D-printed bone model (Fig. 3).

As an outcome, the students were asked to participate in a written assessment a total of three times, which queried knowledge parameters, subjective assessment, and personal motivation (Microsoft Forms, Redmond, WA, Microsoft, 2025). The knowledge questions were designed and reviewed as part of a peer review (3 specialists in orthopedics and trauma surgery). The subjective assessment and personal motivation were determined using modified POSO-E and NASA TLX scores [15, 16]. The first test took place immediately before, and the second directly after the teaching unit. Four weeks later, the students were contacted again via email to complete the same test. A single reminder to complete the test was sent if there was no response after 2 days.

An ethics approval from the Hamburg Ethics Committee was obtained before the start of the study (2024-300559-WF).

### 3D printing

An anatomically accurate model of the distal fibula was generated from anonymized high-resolution CT data of a healthy adult patient using Mimics Innovation Suite 24.0 (Materialise NV, Leuven, Belgium). Segmentation included the full fibular shaft and lateral malleolus, with particular attention to preserving cortical and medullary contours. The 3D mesh was post-processed in Meshmixer 3.5 (Autodesk Inc., USA), where surface smoothing and defect repair were performed. A uniform wall thickness of 2 mm was applied to the outer cortex using hollowing functions, creating a shell suitable for internal filling with structural support material.

**Fig. 4:**
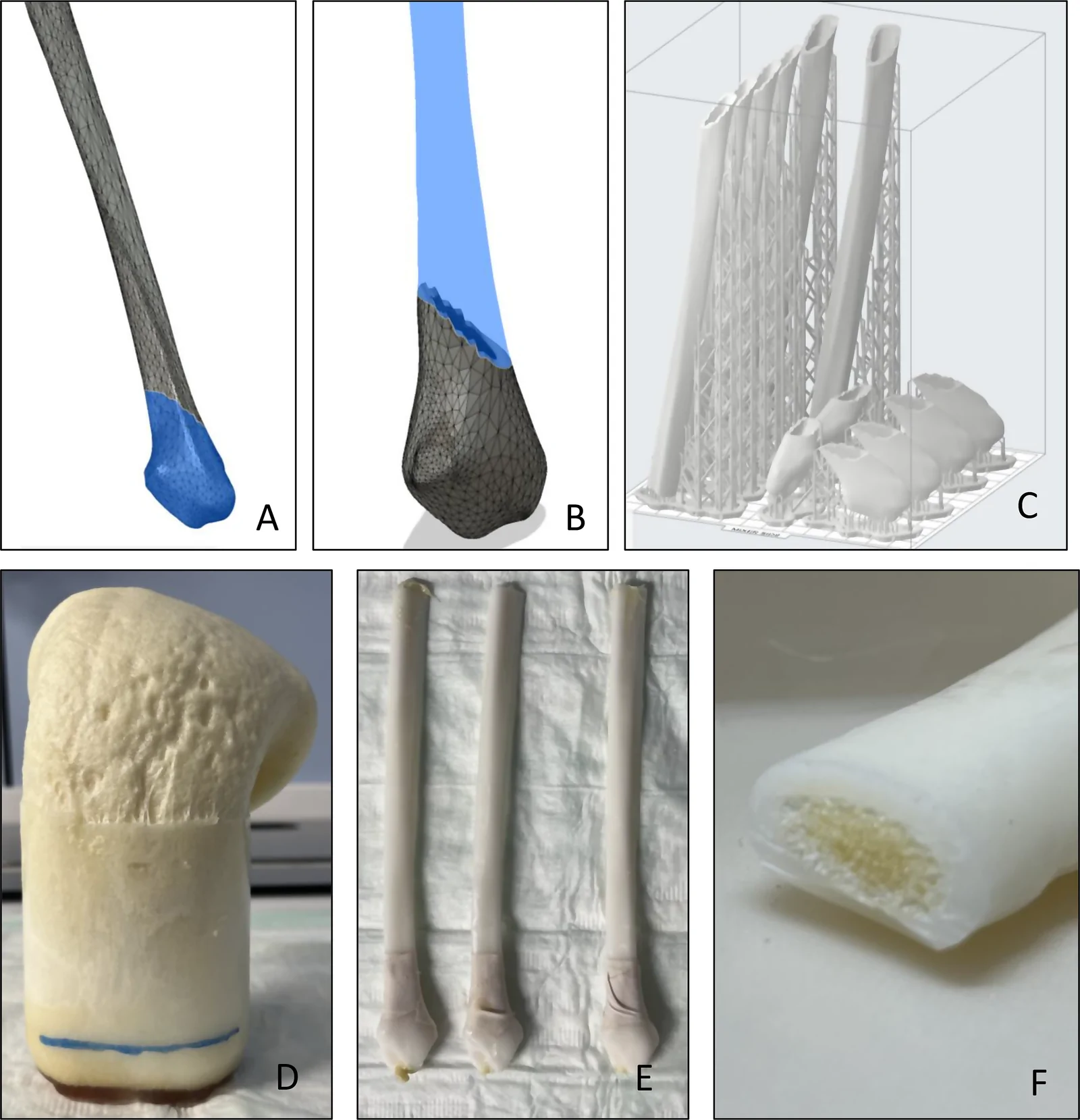
Fracture model of a distal fibula created using Fusion 360 (**A**, **B**); 3D printing of the proximal and distal fracture fragments using Formlabs PreForm software (**C**); internal filling of the 3D-printed models with polyurethane foam (**D**, **E**) (PUR foam, 150 g/L, Beil GmbH, Neuried, Germany); after curing, the foam exhibits a spongiosa-like structure (**F**)

To simulate a realistic training scenario for osteosynthesis, a typical oblique Weber B fracture of the distal fibula was artificially introduced using Fusion 360 (Autodesk Inc.) (Fig. 4 A, B). The fracture line extended from the anterior to the posterior cortex at the metaphyseal-diaphyseal junction. The fractured fibula was digitally separated into two fragments (proximal and distal) and exported as individual STL files.

All models were printed using a Formlabs Form 3B + SLA 3D printer (Formlabs Inc., Somerville, MA, USA) (Fig. 4C). The chosen resin material was Rigid 4 K (Formlabs), a glass-filled photopolymer characterized by high stiffness and dimensional stability. The models were oriented and sliced using PreForm software (v3.30) with a layer thickness of 100 μm and default exposure parameters for the selected resin. Following printing, support structures were manually removed, and models were washed in isopropyl alcohol (IPA, 96%) using the Form Wash system.

Post-curing was performed using the Form Cure system under 405 nm light for 20 min without heat. This curing protocol was deliberately chosen to reduce residual brittleness in the Rigid 4 K prints, which otherwise tend to fracture prematurely under torsional or bending loads during drilling and screw application. After curing, the fracture fragments were manually realigned and temporarily fixed using medical tape to maintain proper positioning during internal filling.

To enhance the biomechanical realism and simulate the cancellous bone core, all models were internally filled with a rigid polyurethane (PU) foam (PUR-Schaum 150 g/L, Beil GmbH, Neuried, Germany) (Fig. 4D). The foam was injected into the hollow cavity through a venting hole located at the proximal metaphysis, allowing complete expansion and hardening under atmospheric pressure. The selected foam density (150 g/L) was chosen based on values reported for trabecular bone density of the distal fibula in prior ex vivo studies and optimized to approximate tactile resistance during manual manipulation [17] (Fig. 4E-F).

**Fig. 5:**
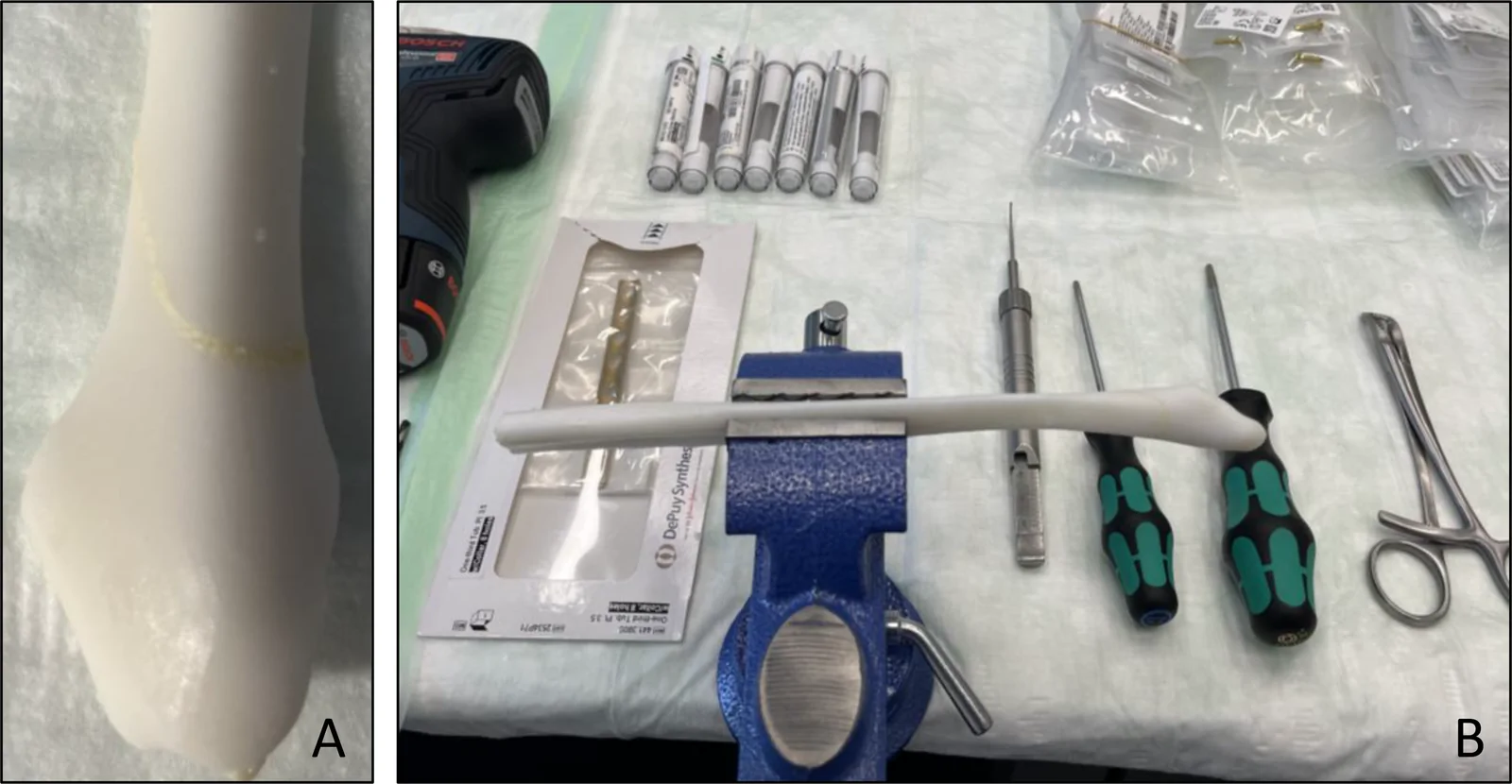
Sealed and filled pre-fractured distal fibula model (**A**); workstation setup for two students performing osteosynthesis of a Weber B fracture (**B**)

The final bonded and foam-filled fibulae were visually inspected for print quality, anatomical fidelity, and fracture line integrity. All models were stored for a minimum of 24 h before use to ensure full curing and mechanical stabilization of both the adhesive and the polyurethane foam. These preparations resulted in stable, reusable bone models suitable for high-frequency use in orthopaedic simulation environments (Fig. 5).

### Statistical analysis

All statistical analyses were conducted using IBM SPSS Statistics (Version 29.0, IBM Corp., Armonk, NY, USA). Statistical significance was defined as p-value < 0.05 (two-tailed) for all analyses. Continuous variables are presented as mean ± standard deviation (SD), and categorical variables as frequencies and percentages. Where appropriate, one-way analysis of variance (ANOVA) analyses were performed at each individual time point to assess between-group differences in performance or subjective ratings. Where significant group effects were detected, LSD (Least Significant Difference) post hoc tests were used for pairwise comparisons between groups. Assumptions for ANOVA were verified by inspecting residual distributions (Q–Q plots) and using the Shapiro-Wilk test for normality. Where violations of sphericity were present, Greenhouse-Geisser corrections were applied. For subjective Likert-scaled items and non-normally distributed outcomes, Kruskal-Wallis tests (between-group) and Friedman tests (within-subject) were used as non-parametric alternatives to validate results. To evaluate the effects of the teaching modality (Group 1: lecture-only, Group 2: model-only, Group 3: lecture + model) across time (t0 = pre-intervention, t1 = immediate post-intervention, t2 = four-week follow-up), repeated-measures ANOVAs (rmANOVA) were performed. These allowed for assessment of within-subject effects (time), between-subject effects (group), and time × group interaction effects on the primary outcomes (knowledge score, processing time, subjective ratings).

## Results

### Processing time analysis

**Fig. 6:**
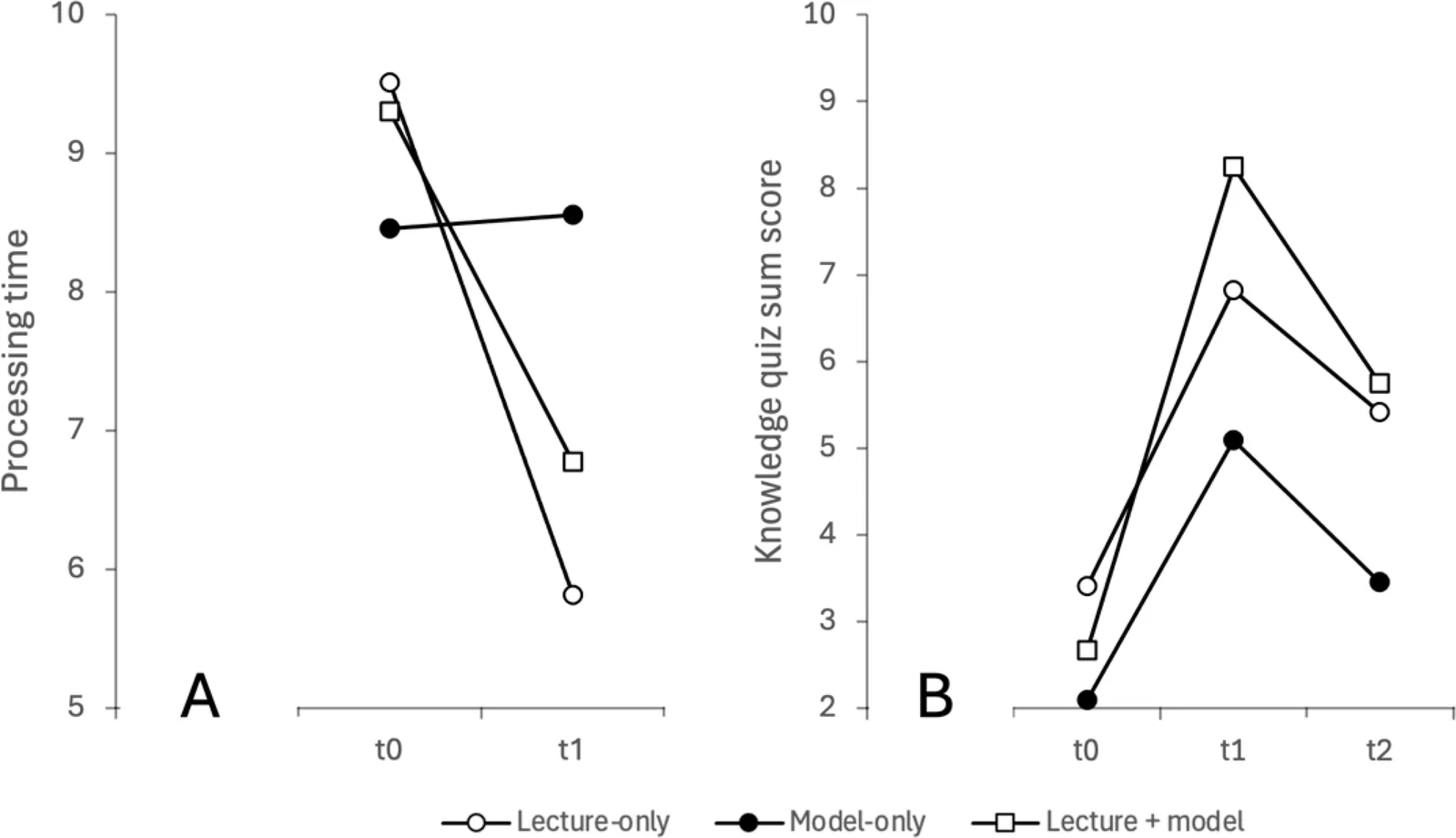
Processing time for the query before (t0) and after (t1) the intervention for all groups (**A**). A statistically significant improvement in processing time was observed in both the lecture-only and lecture + model groups compared to the model-only group (*p* < 0.01). Test performance at all three time points (**B**). A significantly higher performance was detected in the lecture-only and lecture + model groups. This effect was still observable after four weeks (*p* < 0.01)

The analysis of participants’ processing time revealed dynamic shifts in efficiency. Initially, at baseline (t0), a crucial finding was the absence of statistically significant differences in processing time among the three groups. This indicates that all participants started at a comparable level of efficiency, validating the fairness of the initial group assignments (Fig. 6A).

Immediately following the training (t1), significant changes emerged. The lecture-only group recorded the shortest processing time, which was statistically significantly less than the model-based group (*p* < 0.01). This suggests that for the immediate execution of the task, a focused, perhaps more direct, instructional approach led to quicker application. The lecture + model-based group’s processing time fell between the other two, and importantly, there were no statistically significant differences from either the lecture-only or the model-based group, implying that its efficiency was comparable to both, albeit not superior.

Looking at the overall trajectory across all participants, a statistically significant reduction in processing time was observed from t0 to t1 and from t0 to t2. This robust finding confirms that the training, regardless of the specific method, was highly effective in improving task efficiency. While there was an increase in processing time from t1 to t2, this difference was not statistically significant, indicating that the substantial initial improvement largely persisted even after four weeks. This suggests a strong retention of the learned procedural efficiency (data not shown in Fig. 6A).

### Knowledge acquisition assessment

Performance on the knowledge quiz, with a maximum of 11 points, served as a direct measure of factual comprehension. Similar to processing time, baseline knowledge scores (t0) showed no statistically significant differences across the groups, ensuring a level playing field at the study’s outset (*p* = 0.074)(Fig. 6B).

Immediately post-training (t1), a clear hierarchy in knowledge acquisition was established. The lecture + model-based group achieved the highest mean score, which was statistically significantly higher than the model-based group (*p* < 0.01). This compelling result strongly suggests that the synergistic combination of theoretical instruction and practical application leads to the most profound and immediate knowledge gains. Furthermore, the lecture-only group also scored statistically significantly higher than the model-based group, indicating that even a traditional lecture format was more effective for immediate knowledge transfer than solely model-based practice (*p* < 0.05).

Four weeks later (t2), the long-term impact on knowledge retention became evident. Significant group differences in knowledge scores persisted. Specifically, the lecture + model-based group maintained a statistically significantly higher mean score than the model-based group (*p* < 0.05). This highlights the superior long-term retention facilitated by the combined approach compared to a purely practical method, even as overall knowledge showed some decay.

Overall, there was a statistically significant improvement in knowledge from t0 to t1 and from t0 to t2, underscoring the effectiveness of all training methods in boosting knowledge. However, a statistically significant decrease in scores was noted between t1 and t2, indicating an expected pattern of knowledge decay over time. Crucially, the interaction effect between time and group suggests that the pattern of knowledge change over time varied depending on the specific teaching method employed. This implies that different methods not only affect initial learning but also influence how knowledge is retained or lost over time, warranting further investigation into the specific mechanisms at play for each approach (full data shown in Table 1 A).

**Table 1:**
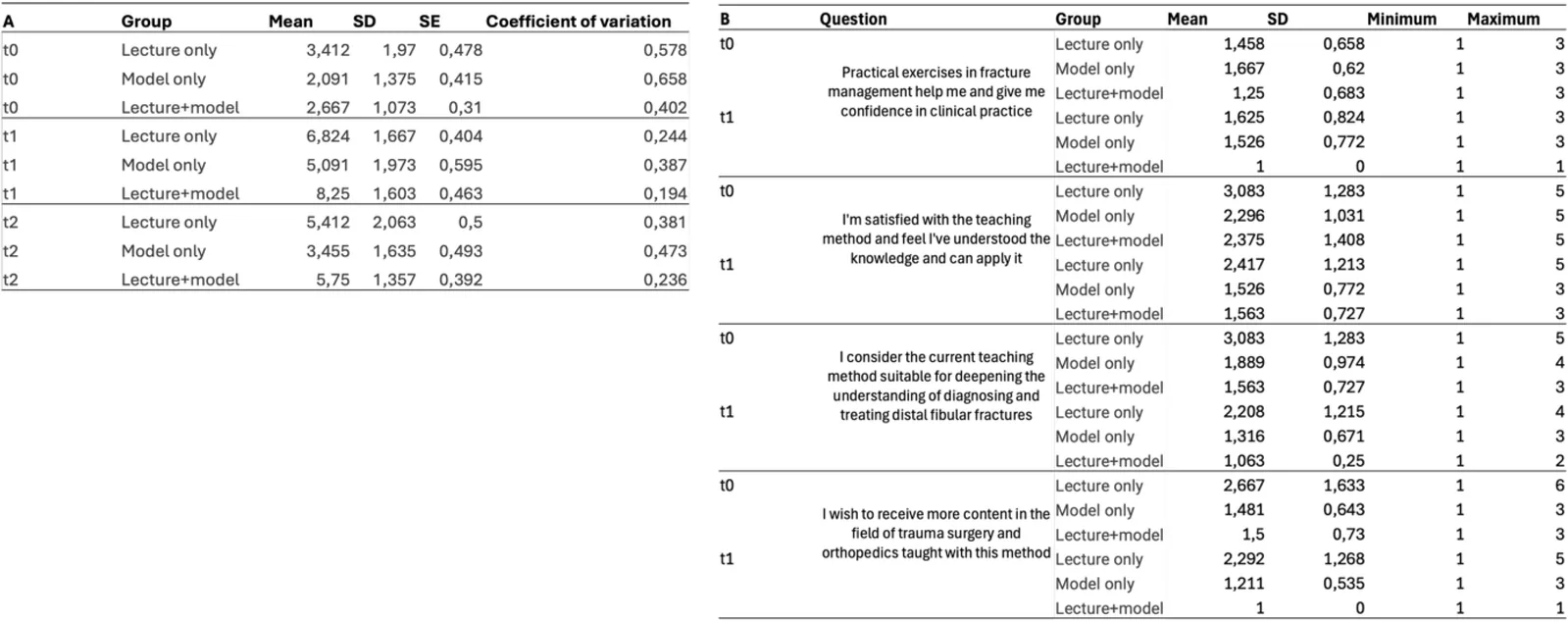
(A) A list of descriptive statistics for the knowledge quiz results at time points t0, t1, and t2 for all three groups. (B) A list of descriptive statistics for satisfaction with the teaching method, based on four select questions using a 6-point Likert scale, for all three groups before and after the intervention (t0 vs. t1)

### Subjective feedback analysis

Participants’ subjective feedback, gathered on a 6-point scale where lower scores denoted stronger agreement, offered valuable insights into their perceptions of the training.

**Fig. 7:**
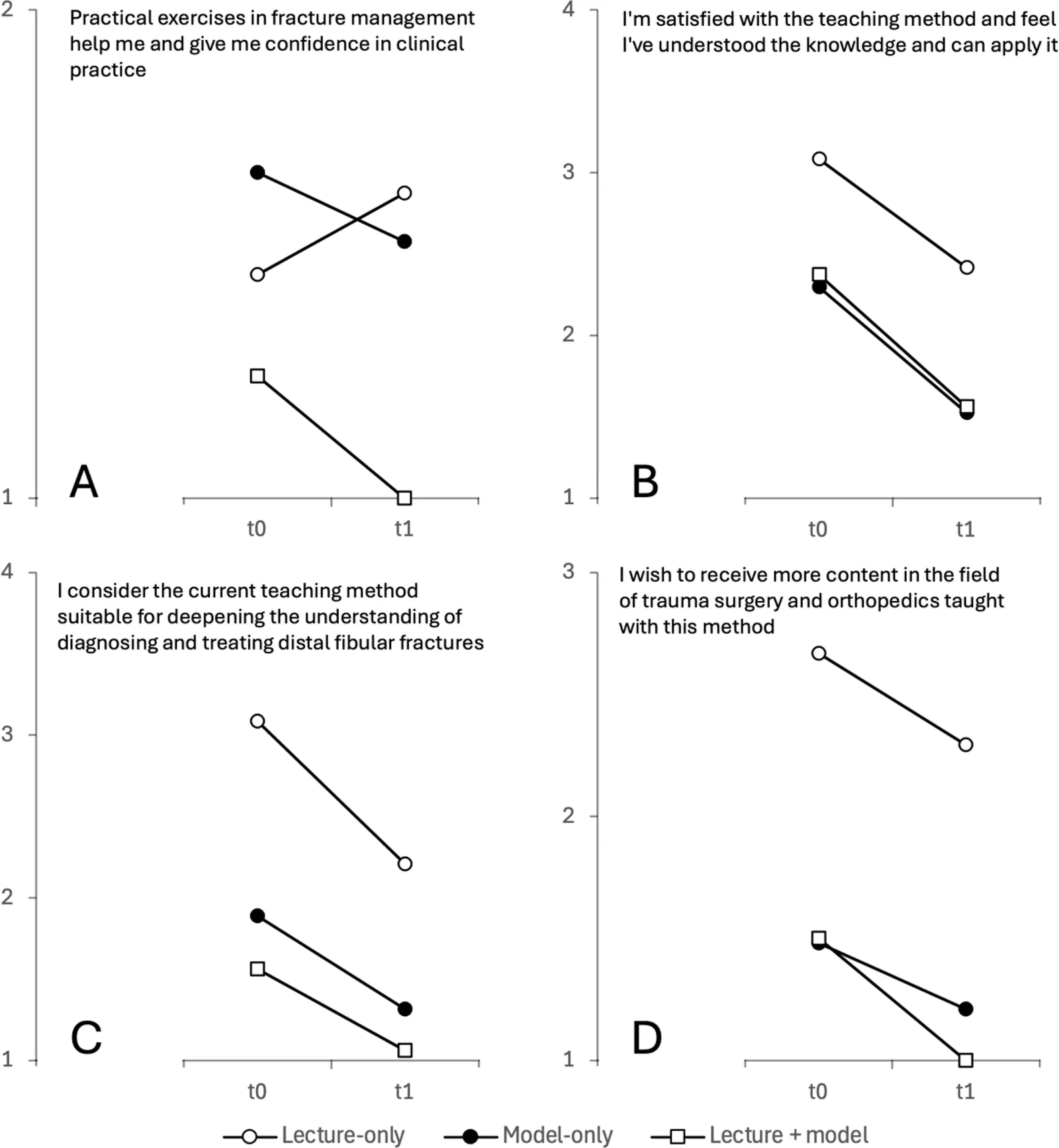
Subjective feedback from the three teaching groups, where a lower numerical rating indicates higher approval. Following the intervention, a significant increase in practical confidence (**A**) was reported by both the model-only and the lecture + model groups. Furthermore, all three groups showed improved ratings across the other measured aspects at t1. Specifically, their satisfaction with the teaching method (**B**), its perceived suitability (**C**), and the desire for more content (**D**) all increased

Regarding the statement “Practical exercises in fracture management help me and give me confidence in clinical practice,” a notable divergence in outcomes was observed between the groups at time point t1. The model-only and lecture + model groups reported an increase in confidence, as indicated by their decreased scores. The lecture + model group, in particular, showed the highest confidence level post-intervention. Conversely, the lecture-only group reported a decrease in confidence from t0 to t1, suggesting that theoretical instruction alone may not be sufficient to build practical self-assurance in this domain (Fig. 7A).

All three instructional approaches led to an increase in student satisfaction and perceived understanding of the subject matter from t0 to t1, as demonstrated by the general downward trend in scores (Fig. 7B). However, the groups that incorporated a practical model (model-only and lecture + model) reported substantially higher satisfaction at t1. Their final scores were nearly identical and markedly lower than that of the lecture-only group, indicating that the hands-on component was a key determinant of satisfaction and the feeling of being able to apply the acquired knowledge.

When evaluating the suitability of the teaching method for understanding the diagnosis and treatment of distal fibular fractures, all groups rated their respective methods more favorably at t1 than at t0. The data clearly indicates a preference for methods involving physical models. The lecture + model group considered their method the most suitable, achieving the lowest score at t1. The model-only group also rated their method as highly suitable, whereas the lecture-only group, despite showing improvement, perceived their method as the least suitable of the three for deepening their understanding (Fig. 7C).

The motivation to receive more content in trauma surgery and orthopedics taught via the same method increased across all three groups from t0 to t1 (Fig. 7D). This suggests a general positive engagement with the educational content. At t1, the desire for more instruction was strongest in the groups that utilized a model. The lecture + model group expressed the highest motivation for future learning, followed closely by the model-only group. The lecture-only group, while more motivated after the session, showed the least interest in receiving more content through that specific format compared to the other two groups (full data shown in Table 1B).

## Discussion

Traditional anatomical training in surgery has relied for centuries on cadaver dissection and didactic lectures as the cornerstones of learning [18]. However, these established methods are facing increasing challenges. These include the limited availability of cadavers [19], ethical considerations, high costs, and the difficulty many students and trainees have in translating two-dimensional representations from textbooks or lecture content into a three-dimensional spatial understanding crucial for surgical planning and execution [20]. In response to these limitations, new technologies, particularly 3D printing, have emerged as innovative tools with the potential to augment and potentially transform anatomy teaching within this field. The literature highlights that 3D printing is considered one of the most relevant new resources due to its pedagogical value and feasibility for orthopedic training [18].

3D technology is now used in many medical fields, including surgical planning, patient education, and especially in medical training. Here, the potential of 3D-printed models is particularly noteworthy for reproducing anatomically complex structures in detail to improve students’ perceptual understanding [21].

Orthopedic training, which deals with complex bone structures and their spatial relationships, presents particular anatomical challenges. The fibula serves as an example of a structure whose complete anatomical understanding – including articulations, bony landmarks and clinical relevance such as fractures – benefits from improved visualization and tactile exploration. Studies have successfully used 3D-printed models for teaching extremity fractures, including tibia-fibula fractures, demonstrating improved knowledge retention and student satisfaction [21]. There are also reports on the printing of fibula models for surgical training simulation.

Although 3D models offer significant advantages, much of the literature positions them more as supplementary tools rather than a complete replacement for traditional methods like dissection or well-structured lectures [18]. The research discussed here, which includes a “combined” group, directly investigates this synergy. The finding that combining different modalities is often advantageous suggests that the true value of 3D models may lie in their thoughtful integration with other teaching methods, rather than viewing them as a standalone solution. Our research, which compares purely theoretical, purely 3D model-based, and combined approaches to fibula surgery, is situated within this evolving landscape. It underscores the need for rigorous comparative studies to determine optimal pedagogical strategies.

Numerous studies report that 3D-printed models lead to improved test results and better knowledge acquisition compared to traditional methods (e.g., 2D images, conventional models) [21]. A meta-analysis showed higher post-training test scores for 3D groups compared to cadaveric or 2D groups [22]. Another study found a pooled effect size of 0.77 for knowledge acquisition with 3D-printed models [23]. Specifically for extremity fractures (including tibia-fibula fractures), 3D models demonstrated advantages in retaining anatomical knowledge [21]. Tangible models can make abstract concepts more concrete, thereby supporting the encoding and retrieval of memory content. Tactile interaction with physical 3D models offers a learning channel distinct from purely visual or auditory inputs. Digital models lack haptic feedback, while physical models enable a tactile response. The literature lists “visual and haptic qualities” as well as “manipulability” as key advantages [24]. Even if a model isn’t perfectly realistic in its texture, the ability to touch and manipulate it offers unique cognitive advantages. This suggests that the physical fibula models used in our study engage a learning modality (haptic) that is absent in purely theoretical learning or learning with 2D images.

Students generally report high levels of satisfaction and engagement when learning with 3D models [19]. They reported having a more positive learning experience. One study reported an estimated effect size of 2.00 for satisfaction scores [23]. Perceived usefulness is also high, with one study finding that instruction with 3D printing was perceived as more useful (RR = 2.29) [22].

This high student satisfaction and engagement are not merely positive side effects [22]. They can lead to increased intrinsic motivation, more time spent on tasks, deeper cognitive processing, and ultimately, improved learning outcomes. Engagement is linked to active participation in learning. Increased interest and motivation are reported, and it’s known that motivation and engagement correlate with academic performance. Therefore, the positive affective responses to 3D models likely contribute causally to better learning, rather than merely correlating with it [20].

Lecture-based learning (LBL) is traditionally described as teacher-centered and often involves direct knowledge transfer [25]. It is recognized that LBL can be effective for imparting foundational knowledge, but it can hinder problem-solving skills, analytical abilities, and reduce student interest/motivation. In the literature, there are cases where 3D models alone have proven highly effective. One study reported that students who exclusively used 3D models achieved statistically superior performance in a post-test compared to a group that only used cadavers and a group that used combined materials for heart anatomy. This is a key finding regarding the potential of a pure model approach. This study is also cited elsewhere, where the pure 3D model group performed best in heart anatomy (p = 0.012) compared to cadaveric material or combined groups [26]. It was noted that it was “difficult to explain why the ‘combined’ group did not provide the same benefit in this specific study.” The potential for self-directed learning and discovery when students interact exclusively with models is also discussed. Haptic interaction and direct manipulation are crucial advantages here [24].

The effectiveness of a pure model approach (Group 2) could heavily depend on the students’ baseline knowledge. Students with prior knowledge (even if informal) might benefit more from a purely model-based exploration than complete novices.

A study found that a 3D-printed model for extremity fractures combined with a lecture significantly improved teaching outcomes compared to a traditional fixed model with a lecture [21]. This study is highly relevant as it involves bone fractures and a similar combined approach. A meta-analysis revealed that 3D visualization + Problem-Based Learning (PBL) was significantly superior to traditional Lecture-Based Learning (LBL) in terms of theoretical knowledge, practical skills, understanding of anatomical structures, and student satisfaction in orthopedic education [25].

Even though PBL differs from a standard lecture, the 3D visualization component is crucial. It was also found that a combined approach (digital and physical models) optimized anatomy education by catering to different learning preferences, improving accessibility, and increasing engagement; students in the combined group showed higher motivation [27]. Another study used a 3D-printed model group that included combined PowerPoint presentations and 3D models and outperformed the pure PowerPoint group in pelvic and spinal anatomy [28]. The rationale is that theory provides foundational context, while the 3D model allows for concrete visualization, manipulation, and application of that theory.

The superiority of combined methods (theory + model) likely stems from creating cognitive resonance, where abstract theoretical knowledge is made concrete and tangible through the model. Purely theoretical instruction can be abstract [25], while 3D models provide concrete, manipulable representations [21]. Combined approaches often lead to superior outcomes [21]. The integration of physical and digital resources accounts for diverse learning preferences and leverages complementary properties [27]. It’s also argued that 2D images offer a theoretical basis, while 3D models provide realism and detail. This suggests that the two modalities reinforce each other; theory provides the “what” and “why,” while the model conveys the “what it looks like/feels like/how it relates.” This dual coding (verbal/abstract + visual/haptic) strengthens learning and memory, creating a richer, more robust mental representation.

The interesting finding that in one study the pure 3D model approach outperformed the combined approach for heart anatomy presents a model paradox [19]. This could occur if the theoretical instruction in the combined group was suboptimal, the model itself was so well-designed and intuitive that it effectively conveyed all necessary information, or if the assessment primarily tested skills best acquired through direct model interaction. This highlights that the quality of each component in a combined approach matters. Simply adding a model to a lecture isn’t a guaranteed improvement; the integration and quality of both components are crucial.

The primary assessment method of our user study is a knowledge quiz. Many studies use pre/post-tests or quizzes to measure knowledge gain. It has been shown that the 3D printing group had advantages in retaining anatomical knowledge for femoral neck and tibia-fibula fractures [21]. A meta-analysis found that 3D groups achieved higher post-training test scores [22]. Another study showed mixed results for extremity fractures (no difference in scores), but better results for the pelvis/spine [28]. This highlights the importance of the specific anatomical region and quiz design. Factors that could influence quiz results include question type and the cognitive level being assessed (recall, application, analysis).

The principle “what gets tested gets learned” is relevant here: The type of knowledge quiz used significantly influences which aspects of learning are captured. Comparative studies on MCQs (Multiple Choice Questions) and VSAQs (Very Short Answer Questions) suggest that VSAQs might better assess applied knowledge and show higher discriminative ability, although they are more difficult [29]. 3D models are intended to improve spatial understanding and applied knowledge. If the user study’s quiz primarily consists of MCQs and assesses simple factual knowledge, it could underestimate the true learning advantage of the 3D model groups (Groups 2 and 3), especially regarding spatial skills, which are difficult to capture with MCQs. Therefore, the design of the quiz itself is a critical methodological consideration that can influence the interpretation of the results.

A meta-analysis found that response times were shorter in 3D printing groups [22]. Shorter test times were observed for the pelvis and spine when using 3D models, but not for the upper/lower extremities [28]. Confidence in understanding is also a relevant outcome. Mixed student comments regarding the difficulty of assessment with 3D models have been noted [30]. The shorter response times reported in some studies with 3D models could indicate more fluent knowledge retrieval or more efficient mental models [22]. This efficiency is a valuable outcome in time-constrained medical curricula and suggests that 3D models might not only improve what students know, but also how efficiently they can retrieve and apply that knowledge.

Constructivism postulates that learners actively construct knowledge through experiences and interactions, rather than passively receiving it [31]. In the context of 3D models, the physical manipulation of models allows students to build their own understanding of anatomical structures and relationships [32]. Kolb’s theory of experiential learning was initially adopted for independent learning with models, but constructivism proved more fitting when active demonstration was required [32]. This is an important topic: pure exploration might require some structuring (a constructivist approach with guidance). The user study’s pure model group might align more with experiential learning if unguided, while the combined group could be more constructivist.

Although pure exploration with a model can be beneficial, the literature suggests that for complex topics, some level of guidance or structuring (a constructivist approach) might be necessary to maximize learning with 3D models. This supports the potential superiority of the user study’s combined group. It is implied that simply giving students a model might not be as effective for anatomy or surgery as providing them with theoretical context or guiding questions to direct their exploration, especially for novices.

Embodied cognition states that cognition is shaped by the body’s interactions with the world [33]. Cognitive integration is supported by interactions with physical models [34]. Haptic learning emphasizes the role of tactile feedback when interacting with 3D models for developing spatial understanding and manual dexterity [24]. Digital models lack haptic feedback, a limitation overcome by physical models [27]. Visual and haptic qualities are highlighted as key advantages of 3D-printed anatomical models (3DPAMs). Handling improves tactile perception and visuospatial awareness. The physical act of holding and manipulating the fibula model can lead to a deeper and more memorable understanding of its form and features than merely viewing images [23].

These pedagogical theories are not mutually exclusive; they often overlap and reinforce each other in the context of learning with 3D models. For example, haptic exploration is a form of experiential learning that facilitates knowledge construction (constructivism), and when the model is well-designed, this occurs by optimizing cognitive load. Physical interaction enables active knowledge construction and this process is made more efficient by reducing unnecessary mental effort.

Cognitive load theory states that learning is optimized when cognitive load is effectively managed. A distinction is made between intrinsic (task complexity), extrinsic (inefficient instructional design), and germane (deep processing) load [35]. In relation to 3D models, these can reduce the extrinsic load required to interpret 2D images and mentally construct 3D structures [27]. It is explicitly stated that extrinsic load can be reduced with 3D models, making them easier to learn from than textbooks. For complex anatomy (such as the articulations of the fibula), well-designed 3D models can make intricate details more accessible, thus helping to manage intrinsic load(27). The dynamic nature of digital models can facilitate the understanding of anatomical variations, thereby helping with intrinsic complexity. By freeing up cognitive resources from extrinsic processing, 3D models can enable students to engage in deeper learning and schema construction (germane load).

Differences in cognitive load generated by the three teaching modalities (theory only, model only, combined) could be a crucial mediating factor explaining differences in quiz performance. Purely theoretical instruction might have a high extrinsic load if students struggle with translating 2D to 3D. A pure model approach might reduce this but could have a high intrinsic load if the model is complex and unguided. A well-designed combined approach could optimize all three types of load by providing theoretical foundations (reducing intrinsic load related to understanding concepts) and a physical model (reducing extrinsic load related to visualization), thereby freeing up capacity for germane load (deeper learning).

The high student satisfaction and engagement with new technologies like 3D models can sometimes be influenced by a novelty effect. It therefore remains an open question to what extent this effect will be sustained longitudinally. The literature on this is inconclusive. In our study, however, a sustained retention of knowledge could be demonstrated over a period of four weeks in the groups that used a combined approach.

## Conclusion

The literature provides strong evidence that 3D models improve anatomical learning, spatial understanding, and student satisfaction. Generally, combined approaches (theory + model) tend to be most effective, though there are specific contexts where pure model approaches are also promising. Pedagogical theories such as constructivism, embodied cognition, and CLT help explain these effects.

The literature largely confirms that 3D models can be effective. The crucial next step for research, including our study, is to delineate how they can best be implemented—for which topics, with which student populations, integrated with what other methods, and in what way assessed. Our study’s three-group comparative design directly addresses important questions in anatomy and surgical education regarding the optimal integration of theoretical and practical learning tools. The focus on a specific anatomical structure (fibula) allows for detailed insights that can complement broader overviews.

## Data Availability

No datasets were generated or analysed during the current study.
